# Exploring bi-directional and SMS messaging for communications between Public Health Agencies and their stakeholders: a qualitative study

**DOI:** 10.1186/s12889-015-1980-2

**Published:** 2015-07-08

**Authors:** Debra Revere, Rebecca Calhoun, Janet Baseman, Mark Oberle

**Affiliations:** Northwest Center for Public Health Practice, School of Public Health, University of Washington, 1107 NE 45th St, Suite 400, Seattle, WA 98105 USA; Department of Epidemiology, School of Public Health, University of Washington, Box 357236, Seattle, WA 98195-7236 USA

## Abstract

**Background:**

Communication technologies that enable bi-directional/two-way communications and cell phone texting (SMS) between public health agencies and their stakeholders may improve public health surveillance, ensure targeted distribution of alerts to hard-to-reach populations, reduce mortality and morbidity in an emergency, and enable a crucial feedback loop between public health agencies and the communities they serve. Building on prior work regarding health care provider preferences for receiving one-way public health communications by email, fax or SMS, we conducted a formative, exploratory study to understand how a bi-directional system and the incorporation of SMS in that system might be used as a strategy to send and receive messages between public health agencies and community-based organizations which serve vulnerable populations, health care providers, and public health workers. Our research question: Under what conditions and/or situations might public health agencies utilize bi-directional and/or SMS messaging for disseminating time-sensitive public health information (alerts, advisories, updates, etc.) to their stakeholders?

**Methods:**

A mixed methods (qualitative and quantitative) study was conducted between April and July 2014. Data collection included a survey distributed to health care providers and semi-structured interviews with providers, community- and government-based organization leaders and directors, and public health agency internal workforce staff. Survey respondents and interviewees were asked about their exposure to public health messages, how these messages are received and how the information in these messages are handled, and in what situations (for example, a local vs. a national event, a pandemic or emergency vs. a health update) a bi-directional and/or SMS messaging system might improve communications between public health agencies and their stakeholder group. Interview and survey data were qualitatively analyzed. Thematic codes were quantitized into dichotomous variables of 0 or 1 on a per respondent basis to enumerate the presence or absence of each thematic code, enable quantitative analysis, and inform interpretation of findings.

**Results:**

Five major themes emerged from synthesizing survey and interview results: 1) Regardless of situational context (emergency vs. non-urgent) and message recipient (stakeholder group), e-mail is a favored modality for receiving public health messages; 2) The decision to use bi-directional, SMS or multiple communication strategies is complex and public health agencies’ need to manage messaging concerns/barriers and benefits for all parties; 3) Both public health agencies and their stakeholders share similar values/uses and concerns regarding two-way public health messaging and SMS; 4) Public health is highly trusted, thus thoughtful, effective messaging will ensure continuation of this goodwill; and 5) Information reciprocity between public health agencies and stakeholders who share their information is essential.

**Conclusions:**

Multiple communication strategies might be utilized but the choice of a specific strategy needs to balance message content (emergency vs. routine communications), delivery (one- vs. two-way), channel (SMS, email, etc.), and public health agency burden with stakeholder preferences and technical capabilities, all while mitigating the risk of message overload and disregard of important communications by recipients.

**Electronic supplementary material:**

The online version of this article (doi:10.1186/s12889-015-1980-2) contains supplementary material, which is available to authorized users.

## Background

Public health agency communications play a central role in minimizing negative outcomes of an emergency, disaster or crisis situation and protecting public safety and welfare [1]. Analyses of recent public health events such as 9/11, the 2001 anthrax attacks, threat of pandemic influenza, extreme weather events such as ice storms, hurricanes and tornados, shootings, and botulism outbreaks, among numerous other examples, consistently reveal gaps in effective communications [2, 3]. Simply broadcasting emergency notifications or instructions is not sufficient; public health agencies need to know that the messages they send are received, effective, informative and actionable. Public health agencies need to know whether their messages are relevant for the recipient, reach the most appropriate and targeted groups at risk for injury or death, and are delivered through a modality and device accessible to target populations [4].

Disseminating time-sensitive, targeted messages can be challenging for public health systems. Currently, public health messaging systems are uni-directional, i.e., messages are sent, typically by email or fax, but there is no mechanism for public health agencies to receive a return reply or acknowledgement from the recipient to document that the message has been delivered. However, new information and communication technologies have the potential to enable bi-directional/two-way communications between public health agencies and their stakeholders—health care providers, community-based organizations serving vulnerable populations, the general public, the agency’s internal workforce—and hold the potential to reduce mortality and morbidity in an emergency, improve public health surveillance during outbreaks, ensure targeted dissemination of alerts to hard-to-reach populations, avert acute events, and enable a crucial feedback loop between public health agencies and the communities they serve [5–8].

Public health emergency message recipients differ in their message delivery modality preferences and needs [9] and these differences may impact message comprehension, source trust, and relevance [10–13]. In our prior work we found that health care providers had an overall preference for receiving public health alerts and advisories by email, as compared to by fax or SMS [9]. However, we also found that prior exposure to communication channel was associated with an increased preference for that channel; i.e., greater familiarity, and possibly comfort, with SMS raised the likelihood of preferring SMS. As cell phones, nearly all of which are text-capable, become ubiquitous in the United States [14], this greater exposure and comfort raise the possibility of disseminating time-sensitive information by SMS regardless of the location of the message recipient. In an emergency SMS is more reliable and stable when compared to voice transmission [15], and costs to public health agencies for receiving messages can be lower [10]. Our prior work with health care providers is limited to one-way communications and only one public health stakeholder group and little is known about bi-directional and/or SMS communication needs, preferences and uses of key stakeholder groups. For example, it is unknown whether clinicians might respond to a public health agency request regarding number of new patients with measles seen in their practice in a given week or whether SMS messages received by community-based organizations will be passed on to their constituents and, if they are, in what format.

The decision to change or expand to a bi-directional messaging system that uses SMS, either in addition to or to replace current communication modalities, cannot be made before investigating the communication practices of public health agencies’ stakeholders. One approach to gaining insights regarding how a new system might be used before investing in its development and implementation is to develop use cases. A use case is a written description of how a system works, represented as a sequence of simple, discrete steps, beginning with the user’s goal and ending with the user attaining that goal [16]. Use cases serve two critical purposes: 1) capturing and defining the requirements, attributes and functions of a system from a user’s perspective and 2) informing realistic scenarios that describe how a user interacts with the system under different conditions. The scenarios described in a use case and its accompanying steps conceptually model how users may expect a system to respond under different conditions. In addition, they can help inform user acceptance and adoption of the new system as well as later testing, expansion and evaluation of the system.

To better understand how a bi-directional system and incorporation of SMS into that system might be used as a public health messaging strategy, we sought to develop a set of use cases built by information elicited from key stakeholders—health care providers, leaders and directors of community- and government-based organizations, and public health agency internal workers—regarding the capabilities, uses, preferences and logistics for sending and receiving public health emergency messages using traditional (email, fax, phone) and novel (SMS) communication modalities. In addition, we sought to increase the evidence base upon which public health agencies make decisions regarding adoption of new technologies and messaging capabilities in support of public health preparedness and emergency response, training and recovery communications.

### Research question

We sought to answer the following research question:

- Under what conditions and/or situations might public health agencies utilize bi-directional and/or SMS messaging for disseminating time-sensitive public health information (alerts, advisories, updates, etc.) to their stakeholders?

In this paper we report the results of our investigation using semi-structured interviews and a survey regarding the current and potential communications practices of target audiences for receiving information from public health agencies during all phases of an emergency: mitigation, response, recovery, and preparedness. The use cases developed from these results will be published in a separate manuscript.

## Methods

### Summary

A mixed methods (qualitative and quantitative) study utilizing a concurrent design was conducted between April and July 2014. In this paper we report the results of our investigation using semi-structured interviews and a survey regarding the current and potential communications practices of key stakeholder groups, selected given their interfacing with public health agencies at local, state, and/or national levels and their service and potential redistribution of public health information to patients, constituents and diverse populations. Three specific stakeholder groups were selected:

- HCPs: Health care providers, selected given their interface with patients and their role in providing care during all phases of a public health emergency.
- CBOs: Leaders and directors of community- and government-based organizations were selected given their role in community emergency preparedness and response and interfacing with their constituents who represent diverse, vulnerable, and hard-to-reach populations.
- PHAs: Public health agency workers, selected given their role in public health emergency communications, response and preparedness.

Data collection included semi-structured interviews (HCPs, CBOs, PHAs) in the state of Montana and in Kitsap and King Counties in Washington State (WA) and a survey of HCPs in King County, WA. Interview and survey data were qualitatively analyzed and thematic codes were quantitized into dichotomous variables of 0 or 1 on a per respondent basis in order to enumerate the presence or absence of each thematic code, enable quantitative analysis and inform interpretation of findings.

### Ethics

The study received approval (Minimal Risk Category 7) from the University of Washington Institutional Review Board.

### Settings

The study was conducted in partnership with three public health agencies: Montana Department of Public Health & Human Services (MT); Kitsap Public Health District (Kitsap County, WA); and Public Health - Seattle & King County (King County, WA). The sites were chosen to represent a range of public health agency organization, messaging system infrastructure and operations, and population densities and demographics. A liaison involved with communications and/or emergency preparedness at each site assisted with subject recruitment.

### Sample size, recruitment and enrollment

We sought to enroll 3–6 subjects in each stakeholder group—HCP, CBO, and PHA—at each site (maximum N = 54). Project liaisons created a list of HCPs, CBOs, and PHAs for recruitment. Recruitment list numbers per stakeholder group for each site ranged between 7–20 individuals and invitations were issued twice by email. The Kitsap County liaison opted to send invitations to interview directly to their list with instructions to contact the study coordinator to schedule; emails were sent to 22 HCPs, 14 CBOs, and 8 PHAs. The MT site opted to send the list to the study coordinator to schedule; emails were sent to 7 HCPs, 24 CBOs, and 10 PHAs. The King County site opted to direct email 4 selected CBOs and to use its HCP and PHA listservs to broadly announce the study interview with instructions to contact the study coordinator to schedule. The latter process resulted in a larger number of interested HCPs than possible to interview for the King County site; 36 HCPs who could not be interviewed were invited to participate by completing an online survey. Table 1 in “Results” details study enrollment.

**Table 1:** Sample demographics

	Interview respondents	Survey respondents	Rural settings	Urban settings
PHAs	8	0	6	2
CBOs	8	1	4	5
HCPs	9	19	5	23

Key: PHA = Public Health Agency Workforce; CBOs = Community-based Organizations; HCPs = Health care providers

### Instrument development

A semi-structured interview was designed to cover the following: current communications practices regarding how public health messages are received and forwarded in the workplace; perceptions regarding feasibility and utility of bi-directional communications with public health agencies, including preferred messaging formats and concerns regarding two-way messages; preferences for receiving time-sensitive or emergency vs. non-urgent public health messages; and use of, utility of, feasibility of and concerns regarding SMS for public health communications. See Additional file 1 for details regarding interview questions. Pilot testing of interviews was conducted internally to streamline questions, remove redundancies, and calculate timing. The final interview guide contained 16 questions and took approximately 30–40 minutes to complete.

A survey version of the interview guide was designed and implemented online for distribution to the HCPs we were unable to interview. The survey contained drop-down menus of most frequently mentioned interview responses and included free text options for all items. See Additional file 2 for details regarding survey questions.

### Data collection

Study participation was voluntary. All interview subjects verbally consented to the study; survey subjects electronically consented to the study. Between April and July 2014, interviews were conducted with CBOs (n = 8), HCPs (n = 9) and PHs (n = 8) at the study sites. The online survey was open between June 15–30, 2014; 20 HCPs completed the survey (1 HCP was a CBO director so these responses were re-categorized as CBO).

Interviews were digitally recorded and transcribed verbatim for import into the atlas.ti qualitative data analysis (QDA) software [17] which facilitates coding, analysis, identification of code co-occurrences or associations, and thematic frequencies. Survey results were segregated into separate documents for each respondent and imported into the QDA program for analysis.

### Coding/Analysis

One coder (BC) thematically coded the interview transcripts and built the preliminary codebook. A second coder (DR) reviewed the coding and codebook. Both coders met to discuss discrepancies, reconcile differences to establish intercoder reliability, and revise the codebook as per standard QDA protocols [18]. During this process, it became apparent that the PHA interviews required separate coding from other stakeholder groups and the codebook was adjusted to reflect this context. The second coder then coded both the interview and survey documents independently. Both coders met to review and reconcile differences and finalize the coding and codebook.

Results were then summarized using enumeration processes and searching for relationships in the data as per QDA and mixed methods protocols [18–20], involving the following steps:

- Codes were collated into themes to qualitatively summarize current communications practices, perceptions regarding bi-directional and SMS public health communications, and emergency vs. non-urgent public health message communication preferences [18, 19];
- Associations between concepts and their context (stakeholder group; rural vs. urban setting) were determined by calculating code co-occurrence frequencies [19, 21];
- Qualitative codes were transformed into dichotomous variables of 0 or 1 on a per respondent basis in order to enumerate the presence or absence of each thematic code and explore its saliency and typicality, i.e., the centrality of the theme [19, 20, 22].

## Results

Table 1 describes the study sample.

### Summary

Five major themes emerged from synthesizing survey and interview results:

- **Regardless of situation, email is a favored communication modality**: Overall, for both emergency and non-urgent situations, email is a preferred strategy for receiving public health messages due to its familiarity, pervasiveness, ease for forwarding, unlimited message length, and perceived permanence.
- **Use of bi-directional and/or SMS communications depends on the situation**: The decision to use traditional, novel or multiple communication strategies—i.e., delivery method (email, SMS, etc.) and directionality (one- vs. two-way)—is complex and dependent on the situation (emergency vs. non-urgent) and message recipient (stakeholder group), as well as the need to manage messaging concerns/barriers and benefits for all parties.
- **Bi-directional messaging has perceived benefits and challenges**: Both public health agencies and their stakeholders share similar values/uses for two-way messaging—case counts; message receipt confirmation; surveillance; surge capacity—and concerns regarding bi-directional messaging—burden; management; technology; privacy, security or HIPAA (Health Insurance Portability and Accountability Act) considerations; concerns regarding information utility, use and potential for misunderstanding replies.
- **Use of SMS is perceived as potentially beneficial as well as challenging**: Both public health agencies and their stakeholders share similar values/uses for—emergency and post-disaster “eyes on the ground” reports; alternative when phone lines are out of service; short polls; post-disaster check-in of status and availability—and concerns regarding SMS for messages—receiving SMS on personal phones; restrictive screen space; SMS is not persistent and easy to ignore; limited cell coverage; security; and inability to forward.
- **Public health agencies are highly trusted by their stakeholders**: Public health agencies are perceived positively, as trustworthy and credible by their stakeholders; thoughtful, effective messaging that does not overwhelm recipients will ensure the continuation of this goodwill.
- **Information reciprocity between public health agencies and their stakeholders is needed**: While free flow of information is desirable to both public health agencies and their stakeholders, requests for stakeholder information must be perceived as necessary/critical and public health agencies need to engage in information reciprocity—i.e., sharing results generated by information submitted by stakeholders to demonstrate its utility and value and ensure these results can be utilized by stakeholders in their own work and will be used by public health agencies to improve a community’s health.

Details regarding these themes follows. Because PHA workforce coding and analysis were segregated we report these results separately from CBO and HCP stakeholder results. In addition, due to some differences in administration of interview vs. survey questions, some results are reported separately.

### Results: Public health workforce

Eight interviews were conducted with PHAs (Kitsap County, n = 3; MT, n = 3; King County, n = 2) working in the areas of communicable disease, emergency preparedness, nursing, administration, environmental health, and epidemiology.

#### Current communication practices

All PHA interviewees communicate regularly with outside constituents, sending public health alerts, advisories, and updates using a variety of modalities, including social media (Facebook). While all use email to communicate with their stakeholders, only one rural public health agency uses email alone (Table 2A). In an emergency situation, such as extreme weather which can close a public health agency, workforce communication is accomplished using a variety of modalities (Table 2B), including SMS. While most of the respondents use text personally, all reported SMS is used in the workplace for different purposes (Table 2C): to reach patients served by the public health agency for appointment reminders; for professional communications with colleagues; and, very occasionally, with stakeholders such as small groups of health care providers. No one reported using SMS to send mass communications.

**Table 2:** Public health agency workforce communications

A. Public health agency communicates with its stakeholders using:					
	Email only	email + fax	email + social media	email + fax + social media	email + social media + SMS
Rural (n = 6)	1	3	0	2	0
Urban (n = 2)	0	0	1	0	0
B. In an emergency, public health agency communicates with its workforce using:					
	Call/phone only	email + call/phone	email + SMS	email + SMS + phone	
Rural (n = 6)	1	1	3	1	
Urban (n = 2)	0	0	1	1	
C. SMS is used in the workplace to communicate with:					
	colleagues	patients	stakeholders	colleagues + patients	colleagues + stakeholders
Rural (n = 6)	2	1	1	2	1
Urban (n = 2)	0	1	0	0	1

#### Bi-directional communications: utility and concerns

The perception of the PHA group is that all messages have the potential for two-way communication since they include a phone number or email address to which recipients can respond with questions or concerns. However, most reported that an alert message is intended as a one-way sharing of information. The exception was one rural PHA interviewee who reported that all messages require a response from recipients and follow-up calls are made if there is no confirmation of receipt.

When asked to articulate the usefulness of two-way messaging, respondents identified different uses: message receipt confirmation of critical communications; surveillance purposes, such as to rapidly collect disease-specific data; and surge capacity information, such as a hospital’s status after an event such as earthquake. The most commonly cited use was case counts:

*“If you are sending out a case definition to providers, then you could get a very quick case count. Get rapid demographics and work out the rest later. You couldn’t get a case report this way because it is not secure but you could get a quick count.” (Participant 14, PHA*)

The following groups or stakeholders were identified as those public health agencies would be more likely to send a two-way (as opposed to one-way) message to: health care providers, dental profession, pharmacies and pharmacists, homeless and women’s shelters, school nurses, emergency management response, and tribal groups. However, concern was expressed that these valuable partners could be overwhelmed or desensitized to communications:

*“You know, you could overdo and there would be information fatigue. If you send too many (2-way messages) then you can see people just delete without reading the thing.” (Participant 17, PHA)*

*“I also think it is easy to burn out the public or providers, so being judicious is important. You want to send only important information.” (Participant 20, PHA)*

Respondents expressed concern regarding management of reply communications in a two-way messaging system. Overall, there was agreement that a response should only be sought when there was a real and immediate need for the data and a clear plan for managing and utilizing the replies.

*“We’d have to come up with a system to use to manage those replies, the system would be a little skinny after hours.” (Participant 12, PHA)*

The technology needed for a bi-directional messaging system was another concern raised by several respondents, both the challenge of funding the technology and the need to learn how to best utilize this kind of system.

*“Between the state and our jurisdiction there are some issues with technology. We are on really old operating systems so it can be hard to open their messages if they are attachments. So there are technology challenges. You know, a lot of these small jurisdictions are money poor – we just are getting rid of the last computer here that was running Windows 98.” (Participant 14, PHA)*

However, the primary concern regarding bi-directional communications was the staff capacity to handle incoming messages, particularly during an emergency:

*“The problem with (two-way messages) is that when there is a big emergency they may not have the capacity in our communicable disease unit or in environmental health - they can’t take time out of doing whatever else they are trying to do to handle that emergency situation.” (Participant 13, PHA)*

*“When our staff is swamped and can’t analyze information collected through two-way messages. During H1N1 we were so busy that we wouldn’t have had time to review the messages even though it might have been helpful to have the information coming in; we would not have looked at it.” (Participant 6, PHA)*

#### Using SMS for Communications

While most of the participants use text personally, the group was mixed in the use of text for professional purposes. A frequently stated reason for this is the lack of professional hardware for texting: with the exception of one respondent who had access to an agency-owned device, all use their personal phones. All interviewees stated there could be beneficial uses for SMS, primarily for quickly obtaining “eyes on the ground” information in an emergency or as an alternative communication modality when phone lines and other venues are out of service. Other uses include: one-way broadcasting such as Amber Alerts, sending short poll requests, and a tool for reaching public health agency workers who are in the field.

*“I could see that (SMS) would be a great way to do a quick tally of vaccine rates. In 20 years when the older population are texting, finding out what proportion have shingles! Voting style responses. Quick pop surveys. It would be very helpful.” (Participant 20, PHA)*

*“If we need to know a preference [..] like ‘yes, I intend to get the vaccine’ or ‘no, I won’t get the vaccine.’” (Participant 6, PHA)*

However, most respondents stated that SMS should be used in conjunction with other forms of communication in order to maximize its reach:

*“I would want it to fit the demographics. I’d always want to use two modes – phone and text or email and text.” (Participant 17, PHA)*

Challenges identified to using SMS include: limited screen space and character length, uncertainty whether a phone number is personal rather than organizational/professional, and tracking replies coming in via SMS. In addition, respondents expressed concern over whether to use SMS instead of or in addition to other traditional methods of communicating with stakeholders:

*“I would be curious about what groups respond better to texts. We do try to segment how we send messages, so I would want to make sure that they are wanting a text over another form of communication [..] rather than email or fax.” (Participant 6, PHA)*

### Results: Aggregated surveys and Interviews (CBOs and HCPs)

Seventeen interviews were conducted with CBOs (n = 8) and HCPs (n = 9) and 20 surveys were completed (CBO, n = 1; HCP, n = 19). CBOs included managers or directors of programs serving immigrant; elderly/aging; low-income housing; and children, youth and family constituents, as well as community-, county-, regional-level emergency and disaster response programs. HCPs worked in emergency department, general internal, infectious disease, and pediatric medicine in hospitals, ambulatory/outpatient clinics, and specialty service clinics. Two HCPs were nurses, one of whom worked as a school nurse.

#### Current communication practices

Regarding how respondents receive information about public health events in their workplace, 97.3 % (36/37) named a public health agency listserv as their primary source (Table 3A). HCPs also indicated they receive information about public health events through a workplace listserv, colleagues, in-person (such as in meetings), and social media. CBOs also named media and social media as an important source of public health information in addition to the listserv. Of note, although a listserv was named by all rural respondents as their source for public health information, the second most frequently named source was fax.

**Table 3:** CBO & HCP Survey & Interview Respondent Characteristics

	CBOs (n = 9)				HCPs (n = 28)			
Urban (n = 28)	5 (17.9 %)				23 (82.1 %)			
Rural (n = 9)	4 (44.4 %)				5 (55.6 %)			
A. How public health messages/information are received in the workplace^*, †^								
	PH listserv	Work listserv	Phone	Fax	Colleague	(Social) Media	In-person	Other
CBOs (n = 9)	8	0	2	2	2	3	1	0
HCPs (n = 28)	28	8	1	4	6	6	2	1
Urban (n = 28)	27	7	1	1	8	8	1	1
Rural (n = 9)	9	1	2	5	0	1	2	0
B. Do you pass on the PH messages/information you receive in your workplace?^*^								
	Yes (N, %)				No or Did not Discuss (N, %)			
CBOs (n = 9)	9 (100 %)				0			
HCPs (n = 28)	16 (57.1 %)				12 (42.9 %)			
Urban (n = 28)	18 (64.3 %)				10 (35.7 %)			
Rural (n = 9)	7 (77.8 %)				2 (22.2 %)			
C. How and to whom public health messages/information passed on in your workplace?^*, †^								
	Email	Co-workers	Colleagues outside work	in-person	(social) media	Patients/constituents		Phone
CBOs (n = 9)	4	7	8	2	3	4		1
HCPs (n = 16)	11	14	7	6	6	7		0
Urban (n = 18)	13	14	10	7	6	8		1
Rural (n = 7)	2	7	5	1	3	3		0
D. If SMS is used in your workplace, how it is used^*, †^								
	Communications with colleagues		Communications with patients/constituents		Mass employer communications	Do not use SMS in the workplace (N, %)		
CBOs (n = 9)	3		2		1	4 (44.4 %)		
HCPs (n = 28)	10		1		3	13 (46.4 %)		
Urban (n = 28)	9		2		2	13 (46.4 %)		
Rural (n = 9)	4		1		2	4 (44.4 %)		

*Combined responses from interview (n = 17) and survey (n = 20) participants [missing, n = 0]

^†^Multiple answers possible

While all CBO respondents indicated they pass public health information on to others, only 57.1 % of HCPs similarly pass on public health information (Table 3B). Of those respondents who further disseminate the public health information they receive, CBOs primarily pass information on to colleagues outside their workplace (8/9) and to co-workers (7/9) while HCPs primarily pass this information on to co-workers (14/16). Regarding method for passing information on, 4/9 CBOs and 11/16 HCPs use email.

A little more than half of all respondents (55.6 % CBOs, 53.6 % HCPs) report using SMS in the workplace (Table 3D), primarily to communicate with colleagues.

#### Emergency vs. non-urgent communications

When asked to identify the best way to receive time-sensitive or emergency public health messages (Table 4A), overall respondents favored communications by both email and SMS (48.7 %). However, over half of CBOs (55.6 %) preferred emergency communications by SMS only and over half of HCPs (57.1 %) preferred both email and SMS.

**Table 4:** CBO and HCP Messaging Preferences

A. Best way to receive a time-sensitive or emergency message from a public health agency^*^				
	Email (N, %)	SMS (N, %)	Both email and SMS (N, %)	Neither email or SMS ex, prefer phone (N, %)
Total (n = 37)	7 (18.9 %)	11 (29.7 %)	18 (48.7 %)	1 (2.7 %)
CBOs (n = 9)	2 (22.2 %)	5 (55.6 %)	2 (22.2 %)	0 (0)
HCPs (n = 28)	5 (17.9 %)	6 (21.4 %)	16 (57.1 %)	1 (3.6 %)
Urban (n = 28)	4 (14.3 %)	7 (25.0 %)	16 (57.1 %)	1 (3.6 %)
Rural (n = 9)	3 (33.3 %)	4 (44.4 %)	2 (22.2 %)	0 (0)
^*^Combined responses from interview (n = 17) and survey (n = 20) participants [missing, n = 0]				
B. In a non-urgent situation, how would you prefer to receive a public health agency message?^‡^				
	phone call only	email only	email + phone call	email + SMS
CBOs (n = 7)	0	5	1	1
HCPs (n = 6)	0	5	0	1
Urban (n = 7)	0	6	0	1
Rural (n = 6)	0	4	1	1

^‡^Interview (n = 13) item only [missing, n = 4]

*“Texting is more personal and timely. Reserve it for emergencies and when there is something very urgent that needs an immediate response.” (Participant 25, CBO)*

*“Texting is ok for an emergency but health alerts are better via email, so use both.” (Participant 43, HCP)*

*“(Use) email when you have time and it is okay to take the time to share more information and to be more exact. Text would be for ballpark figures that are needed immediately. In the bigger emergencies – or earlier on in the emergency response, text would be great. Later when you want exact figures, then moving over to email makes more sense.” (Participant 23, CBO)*

Interview participants overwhelmingly favor email for receiving non-urgent public health messages (Table 4B). The ease of forwarding and the ability to receive detailed information in this format were cited as the main reasons for this preference (more specifics in “Using SMS for Communications” below).

*“If it is not urgent, like if there is whooping cough in the community – email would be best.” (Participant 2, HCP)*

*“With email I open that when I have time, when I am at my desk and I am choosing to take time to look at email and read it. [..] Texting is hard to forward so sharing that information is then challenging. Email is better for day to day information.” (Participant 25, CBO)*

#### Bi-directional communications: utility and concerns

Among interview respondents, all CBOs and HCPs stated they would be willing to reply to a public health agency request for information (Table 5A).

**Table 5:** Bi-directional Messaging

A. Willingness reply to one- vs. two-way communications from a public health agency^‡^							
	1- vs. 2-way depends on situation			2-way always best		1-way always best	
CBOs (n = 8)	6			2		0	
HCPs (n = 9)				1		1	
Urban (n = 8)	6			1		1	
Rural (n = 9)	7			2		0	
^‡^Interview (n = 17) item only [missing, n = 0]							
B. If a public health agency requests a reply, preferred way to respond^**^							
	Email (N, %)	Email + SMS (N, %)	Email + phone (N, %)	Email + phone + webform (N, %)	Email + webform (N, %)	Email + SMS + webform (N, %)	
CBOs (n = 8)	4 (50.0 %)	0 (0)	0 (0)	3 (37.5 %)	1 (12.5 %)	0 (0)	
HCPs (n = 27)	16 (59.3 %)	3 (11.1 %)	2 (7.4 %)	0 (0)	3 (11.1 %)	3 (11.1 %)	
Urban (n = 26)	15 (57.7 %)	3 (11.5 %)	1 (3.9 %)	1 (3.9 %)	3 (11.5 %)	3 (11.5 %)	
Rural (n = 9)	5 (55.6 %)	0 (0)	1 (11.1 %)	2 (22.2 %)	1 (11.1 %)	0 (0)	
^**^Combined responses from interview (n = 16) and survey (n = 19) participants [missing, n = 2]							
C. Concerns about bi-directional communications ^‡‡,†^							
	Burden to reply	HIPAA/privacy/security	Unsure how info is used	Info could be mis-understood	Unsure info is useful	Concern re who receives reply	NO concerns
CBOs (n = 8)	0	2	0	0	1	8	0
HCPs (n = 21)	3	15	3	2	2	14	3
Urban (n = 20)	0	12	3	2	1	13	3
Rural (n = 9)	3	5	0	0	2	9	0

^‡‡^Combined responses from interview (n = 17) and survey (n = 12) participants [missing, n = 8]

^†^Multiple answers possible

*“I always want the ability to reply!” (Participant 2, HCP)*

*“My philosophy is that we are stronger when we work together so I would always give information if asked.” (Participant 4, CBO)*

*“Certainly if there was a new or unusual case, it would be good to send that. I could see it could be helpful (for public health) to find a pattern and maybe pick up on things sooner.” (Participant 10, HCP)*

*“Yes, we would reply. An example might be when we were operating a severe weather shelter, so we were communicating about people we were serving – sending information about the number of people we had at the shelter. We would definitely respond.” (Participant 23, CBO)*

While there was consensus that respondents *always* want an option of contacting public health agencies for more information if they have questions or concerns about a message, interview respondents also indicated that public health agencies should only request information if there was a clear and immediate need. Otherwise, respondents preferred one-way communications.

*“I would hope that it wouldn’t be asked for and then just something that sits there. We are too busy to do something that is not used.” (Participant 7, CBO)*

*“Yes, if it was a quick and brief question. I guess I would do that if I could just respond quickly – like, yes, I am seeing this in my patients.” (Participant 11, HCP)*

*“I think a one-way message is great if it is just information sharing and they don’t need a response. You only want to ask for a response if you need one.” (Participant 25, CBO)*

*“I would never respond unless there is a really good reason…” (Participant 16, HCP)*

If a public health agency is requesting a reply to a message, 50 % of CBOs and 59.3 % of HCPs (both interview and survey respondents) would prefer to reply by email (Table 5B). Of note, half of the CBO respondents indicated they would prefer to have more than one option for replying, for example email, phone, and a web-based form for submitting responses.

*“Two way communications via email is preferable so that the trail of communications is documented.” (Participant 27, HCP)*

*“I would like to see all the options, if I can make a quick call while I am out then I do that, if I am at a computer I can respond there. All the options are best.” (Participant 3, CBO)*

When asked how they presumed a public health agency might use information they sent, a number of interview participants (11/17) spontaneously made statements of trust regarding public health, for example, expressing their confidence that the public health agency will use the information sent wisely and to improve the community’s health. In addition, several respondents (6/17) stated they would want to be informed how the public health agency used their information.

*“I think (public health is) very quality oriented so I would expect them to use the information that I send in quality improvements or to inform them in some way. They do good work over there.” (Participant 2, HCP)*

*“I would hope (public health) would use it for planning and implementation and using it to track a situation. I would expect some kind of response, you know it would be nice to get information back so you can get a broader picture of the community at large because I mostly have just tunnel vision – I can only see what I can see from here with this perspective.” (Participant 25, CBO)*

*“We assume (public health is) using it in a way that is beneficial to the community [..] for Epi investigations or for planning a response.” (Participant 18, HCP)*

However, several concerns were identified regarding bi-directional communications with public health agencies (Table 5C), including concern about who might be receiving replies; HIPAA, privacy or security concerns, especially with identifiable patient information; burden, i.e., not wanting to spend extra time looking up information in order to formulate a reply; and issues regarding how the information sent might be used, the potential for a public health agency misunderstanding the information, and whether the information sent is useful.

*“Obviously, I would want nothing that could be traced back to a patient. HIPAA compliant. It would have to be really generic.” (Participant 11, HCP)*

*“I wouldn’t be concerned so long as information is public information – of course, client information wouldn’t be shared and we have security and all that but that almost goes without saying. We wouldn’t give our lists or anything like that.” (Participant 7, CBO)*

*“As long as (replying) doesn’t require much work – not counts or look up charts – that, I wouldn’t do.” (Participant 9, HCP)*

*“I think I would send general stuff but no identifiable information.” (Participant 21, HCP)*

#### Using SMS for communications

Several considerations regarding the use of SMS for public health agency communications were identified: concern that SMS is “easy to dismiss” or ignore; limited reception which can make SMS unreliable; hesitations over security; respondents only owning a personal phone; the limited space and character length of SMS being too restrictive; and inability to further disseminate SMS messages (Table 6).

**Table 6:** Concerns about SMS Communications^***,†^

	can’t forward	easily missed	HIPAA/security	limited space	personal phone only	reliability	don’t use SMS
CBOs (n = 7)	4	1	2	1	2	1	2
HCPs (n = 10)	0	3	5	5	1	3	1
Urban (n = 10)	3	2	3	4	1	4	1
Rural (n = 7)	1	2	4	2	2	0	2

^***^Combined responses from interview (n = 15) and survey (n = 2) participants [missing, n = 20]

^†^Multiple answers possible

*“I think the downside with texting is if the phone is not right with you might not hear the text so there could be a delay in response if you don’t hear it [..] so I’m hesitant to use it.” (Participant 15, CBO)*

*“I would open it and close it and then forget about it because I wouldn’t have time at that moment to answer it.” (Participant 11, HCP)*

*“I find texting annoying because it requires an immediate response. I rarely go back through texting.” (Participant 19, HCP)*

*“My phone is frequently dead, at least once a week, so texting is not always a reliable system for me.” (Participant 39, HCP)*

*“I think when more info has to be given the text is small. Specific and detailed instructions would be much better sent via email.” (Participant 18, HCP)*

*“I’m worried about the confidentiality too much to use texting.” (Participant 3, CBO)*

*“I can’t get good reception as we are in a basement office [..] no good service anywhere so texting does not work for me.” (Participant 18, HCP)*

However, although not specifically asked about in the interviews, three HCPs raised the potential for using SMS after a disaster or in an emergency to check-in with their public health agency or to notify the agency of their status and availability:

*“If a reply was requested. Let’s say a disaster or some type thing like that and they wanted to find out if you were operational or had experiences with the disaster - something like that.” (Participant 1, HCP)*

*“In the event of a disaster, using text for organizing people for responding to the emergency, that could be helpful.” (Participant 2, HCP)*

*“I live on Mercer Island and if the bridge is out… public health could use texting to link MDs to other MDs who might need support. For big emergency situations to connect us to other doctors that could help. [..] If there was a blast ‘we need this and this is how you can help’ it would tell me how I could help. To recruit volunteers.” (Participant 21, HCP)*

## Discussion

Rapid advances in communication and information technologies are pushing the public health system and its agencies to meet new challenges to fulfilling its core function of “providing information to the public health community regarding the health of the populations served” [23]. In our study we explored the barriers and facilitators to public health agencies incorporating and engaging with two significant communication and information technology advances—bi-directional communications and SMS—to better understand how these new messaging strategies might be used by public health agencies to communicate with its stakeholders, particularly in emergency situations.

It is not surprising that we found this to be a complex topic. Our findings indicate that multiple communication strategies can be utilized and the choice of a specific strategy needs to balance message content (emergency vs. routine communications), delivery (one- vs. two-way), and channel (SMS, email, etc.) with stakeholder preferences and technical capabilities, all while mitigating the risk of message overload and over-looking of important communications by recipients. Requiring public health agencies to maintain multiple communication systems and match message format both situation and recipient preferences could create an unrealistic burden. However, our study narrows down formats to two primary modes—email and SMS. While email was overall clearly preferred for receiving public health messages, in an emergency or urgent situation, nearly half (48.7 %) of respondents wanted both email and SMS to ensure they received critical messages. Each mode has its disadvantages which can be overcome by the other. For example, email is nearly ubiquitous in the workplace, easily forwarded, unconstrained in message length, and perceived as being persistent and easily referenced or returned to when needed. However, in an emergency, electricity and Internet connections may go down, making it impossible to access email messages. SMS is similarly as ubiquitous as cell phones, but limited in message length, difficult to forward, and perceived as transient. However, in an emergency, cell service is often more reliable, and SMS offers the potential for conducting short polls and obtaining check-in status and updates during and after a disaster.

For public health agency communications systems, both SMS and email involve operational advantages and drawbacks. Both require “opting in” to their service and the burden of updating contact information rests on the recipient. It could be dangerous to the public’s safety for public health agencies to rely solely on email or SMS as, without the resources to handle continuous listserv maintenance and updating, critical messages may not be delivered. Adopting a system to deliver SMS messages also would require opting in and updating challenges while also introducing concerns about security and the potential for misunderstanding messages due to SMS’ limited character length. However, given the two modes complement one another well, particularly in an emergency, utilizing both may be the most effective approach to distribute time-sensitive messages. It is clear research to determine the best combination for different groups of stakeholders that also takes into account whether and how stakeholders further disseminate the messages they receive will enable public health communications to utilize new communication and information technologies to their full potential.

Interactive, bi-directional messaging introduces its own challenges. While it is clear that public health stakeholders in this study trust the public health system, two-way communications introduce the risk of violating that trust if not used carefully. The goal of improving situational awareness by engaging stakeholders in real-time surveillance is a shared objective. But stakeholders must be assured that the data requested is worth submitting by public health agencies returning their analyses or translation of that data into information that is meaningful to stakeholders. Doing so would ameliorate stakeholder concerns regarding the burden of replying, privacy or security, and potential for misunderstanding replies. Information reciprocity between a public health agency and its stakeholders is a standard component of surveillance [24] and should be a key objective when considering adoption of a bi-directional messaging system. Stakeholders need to know overcoming their concerns and barriers is worthwhile because they reap benefits. Our findings indicate that some stakeholders are unsure who would receive their reply and where the information goes within a public health agency. While public health messages always include an email and/or phone number, it may be advisable for public health 2-way messages to include the specific contact person receiving replies to assure recipients that their information is not languishing in a mailbox.

### Limitations

A limitation of this study is the absence of any employees who are responsible for the technologies needed to establish a bi-directional and/or SMS communication system in a public health agency. It is possible that some of the perceived technological barriers to utilizing such a system may be more a reflection of lack of familiarity with novel communication strategies. Another limitation is the representativeness of the sample: HCPs, specifically MDs, were over-represented while CBO stakeholders were under-represented. A final limitation is the potential of introducing bias into the results given our reliance on site liaisons to provide recruitment lists.

## Conclusions

In this paper we report a formative, exploratory study of the current and potential communications practices of public health stakeholders and the public health workforce with respect to bi-directional communications and the incorporation of SMS as public health messaging strategies. This study provides the foundation for conducting a larger investigation in which time-sensitive messages may be sent to public health stakeholders to determine the feasibility of communicating in these different modes or combination of modes. While further investigation is needed, our study is another step in building the evidence base upon which public health agencies make decisions regarding adoption of new technologies and messaging capabilities in support of public health preparedness and emergency response, training and recovery communications.

## Additional files

Additional file 1:
**Interview Guide, PHA, HCP, CBO.**
Additional file 2:
**Survey Items -- HCP.**
