# Supplementary material for: Exploring bi-directional and SMS messaging for communications between Public Health Agencies and their stakeholders: a qualitative study
Source: BMC Public Health. 2015 Jul 8;15:621. doi: 10.1186/s12889-015-1980-2 (PMC4494811; doi:10.1186/s12889-015-1980-2)
Supplement: Additional file 1: — Interview Guide, PHA, HCP, CBO. [file 12889_2015_1980_MOESM1_ESM.docx]

**Appendix. Supplemental Material 1: Interview Guide, PHA, HCP, CBO**

| **Part 1. Current Protocols for Handling Public Health Messages** | |
| --- | --- |
| **Intro:** I'd like to start by learning a little about your role and responsibilities and how public health messages are handled at your workplace. | |
| **Public health agency informants (PHAs)** | **Non-PHAs (HCPs, CBOs)** |
| A. Could you briefly tell me a bit about your position or role in [*NAME OF PH AGENCY*]? | A. Could you briefly tell me a bit about [*NAME OF ORGANIZATION/CLINIC*] and your position or role in that organization? |
| B. In the past 6 months, has [*PH*] issued a public health alert, advisory or, update? [**If YES, probe for topic(s)**] | B. In the past 6 months have you received a message from a local or state public health agency or from the CDC? [**If YES, probe for topic(s)**] |
| C. We're interested in how the decision is made at [*PH*] to send a public health alert, advisory or update. Could you walk me through this process, using the example of a rise in reported influenza-like illness in the community? [**prompts:** What triggers the decision to send a message? Who makes this decision? How are messages sent? To what stakeholder groups? Is delivery tracked? Are there different systems for sending messages?]  Does this process differ if the information is a public health alert, advisory or update? [**probe for details**] | C. We're interested in how [*ORG/CLINIC*] currently receives information about public health events. For example, how would you learn there is a rise in influenza in the community? [**prompts:** How is information about the event received? What kind of information is received? Who sends this information?]  Does this process differ if [*ORG/CLINIC*] receives a public health alert versus an advisory or update? [**If needed, explain difference between a public health alert, advisory, and update**] |
| D. Is [*PH*] also responsible for sending alerts or advisories to other public health organizations? [**If YES, prompt**: What triggers the decision to pass on this information? What kind of information is passed on? To whom? How is the decision to pass on information made and by whom? How is information distributed (email, phone, etc)?] | D. Does [*ORG/CLINIC*] also pass this kind of information on to other organizations or to your constituents/patients? [**If YES, prompt**: What triggers the decision to pass on this information? Who makes this decision? What information is passed on? To whom? How is information distributed (email, phone, etc)?] |

| **Part 2. Bi-Directional Messaging** | |
| --- | --- |
| ***Intro:*** Usually, a public health agency sends one-way messages without expecting a reply. We're interested in learning about situations or conditions when replying to a public health message might be useful. | |
| **Public health organization informants** | **Non-public-health organization informants** |
| Suppose [*PH*] could receive a reply to a public health message... | Suppose [*ORG/CLINIC*] could send a reply to a public health message... |
| A. In what situations might it be useful to receive a reply to a message [*PH*] has sent? [**prompt**: What if the message is an alert? an advisory? updates? How does the decision change if the event is local versus regional or national?] | A. Are there situations in which it might be useful to send a reply to a public health message [*ORG/CLINIC*] has received? [**prompt**: What if the message is an alert? advisory? update? How does the decision change if the event is local versus regional or national?] |
| B. What information would be useful to receive in a reply? [**prompt:** Surveillance? Foodborne illness?] | B. What information would be useful to send in a reply? [prompt: Surveillance? Foodborne illness?] |
| C. Are there specific groups or stakeholders that [*PH*] would be more likely to send two-way messages to? | C. Who would you expect your reply is being sent to? |
| D. How would [*PH*] use the information sent in the reply? [**prompt**: How might a reply be managed at [PH]? Who would need to see the replies?] | D. How might you expect the public health agency to use the information you send in your reply? |
| E. What are some of the challenges in using a two-way messaging system? [**prompts**: undue burden, language limitations, irrelevant or inappropriate responses , etc.] | E. Do you have any concerns about using a two-way messaging system? [**prompts**: undue burden, language limitations, uncertain where reply is sent, etc.] |
| F. During what public health events might it be better to send a one-way message rather than a two-way message? | F. During what public health events might it be better to receive a one-way message rather than a two-way message? |

| **Part 3. Text Messaging and Two-Way Texting** | |
| --- | --- |
| **Intro:** We're interested in learning about your experience with text messaging.[**If needed, provide definition of SMS**] | |
| **Public health organization informants** | **Non-public-health organization informants** |
| A. Is texting used in your workplace? [**If YES, probe**: How is SMS used? Can you give me a recent situation in which SMS was used? **If NO:** Does [*PH*] restrict use of SMS?] | A. Is texting used in your workplace? [**If YES, probe**: How is SMS used? Recent situation in which SMS was used? **If NO** Does [*ORG/CLINIC*] restrict use of SMS?] |
| B. What specific stakeholder groups would you be more likely to communicate with using text and why? [**prompt**: CBOs? HCPs? General public? Other public health agencies? Internally? Local or state government groups? Others?] | B. Would text messaging be a useful way for [*ORG/CLINIC*] to communicate with other organizations or your constituents/patients? |
| C. During what public health events might it be better to not send SMS, but instead use email or the phone or some other medium? | D. During what public health events might it be better to get a public health message via email or phone or some other medium other than text messages? |
| D. Would two-way SMS be useful for ongoing routine communication with stakeholders or only for special circumstances? Or could it be both?[**probe reasons and circumstances**] | D. Would two-way SMS be useful for ongoing routine communication with other organizations your constituents/patients, and public health? Or would text messages only be useful for special circumstances? [**probe reasons and circumstances**] |
