# Supplementary material for: Exploring bi-directional and SMS messaging for communications between Public Health Agencies and their stakeholders: a qualitative study
Source: BMC Public Health. 2015 Jul 8;15:621. doi: 10.1186/s12889-015-1980-2 (PMC4494811; doi:10.1186/s12889-015-1980-2)
Supplement: Additional file 2: — Survey Items -- HCP. [file 12889_2015_1980_MOESM2_ESM.docx]

**Appendix. Supplemental Material 2: Survey Items -- HCP**

1. Please select the one setting that best describes your work place:

□ Community Clinic

□ Community-based Organization

□ Emergency Room or Emergency Department

□ First Responder/Emergency Services

□ Hospital

□ Lab

□ Outpatient Clinic

□ Private Practice / Office

□ Public Health Agency

□ Public Health Clinic

□ Public Health Lab

□ Retail Pharmacy

□ School Clinic

□ Urgent Care Clinic

□ Veterinary Clinic

□ Other:

2. What is your position or work role? Select all that apply.

□ Administration

□ Infection Control

□ Laboratory

□ Management

□ Nurse

□ Nurse Practitioner

□ Pharmacist

□ Physician

□ Physician Assistant

□ Veterinarian

□ Other:

3. We're interested in how you currently receive information at work about issues of public health importance. For example, how would you learn that there is a rise in influenza or measles in the community? Select all that apply.

□ Public health email/listserv

□ Fax from public health

□ Information forwarded or passed on through workplace email/listserv

□ Information forwarded or passed on by a colleague

□ Text message or SMS

□ Information from media source (radio, TV, facebook)

□ Other:

4. In the past 6 months have you received any message from a public health agency (local public health agency, state health department, CDC)?

□ Yes

□ No (SKIP TO 8)

5. How did you receive the message?

□ Fax

□ Forwarded or passed on by a colleague

□ Forwarded or passed on through work place email/listserv

□ Phone

□ Public health email/listserv

□ Social media feed (facebook, Twitter)

□ Text message or SMS

□ Other:

6. When you received this message, did you pass any of the information on to other organizations or to your patients or clients?

□ Yes

□ No (SKIP TO 8)

7. How did you pass this information on? Please check all that apply:

□ Email, internally to colleague(s) or others in your workplace

□ Email, externally to colleague(s) outside your workplace

□ Email to patient(s) or client(s)

□ Fax

□ In person, to colleague(s) or others in your workplace

□ In person, to colleague(s) outside your □ workplace

□ In person, to patient(s) or client(s)

□ Phone

□ Posted to a social media or blog account or website

□ Text message

□ Other:

8. Usually, a public health agency sends one-way messages without expecting a reply. We're interested in learning about situations or conditions when replying to a public health message might be useful. Are there situations (emergency, pandemic outbreak, etc.) in which it might be useful to send a reply to a public health message? [OPEN-ENDED]

9. If you were asked to reply to a public health message, how would you prefer to send your reply? Select all that apply.

□ Email

□ Fax

□ Fillable form on a website

□ Phone

□ Text message

□ Other:

9. If public health agencies were able to set up a two-way system so you could send replies to their

messages, would you have any concerns about a system like this? Select all that apply.

□ Uncertain who is receiving my reply

□ Uncertain how information I send will be used

□ Message security concerns

□ Privacy concerns about sending personal health information

□ Undue burden of extra communications

□ Uncertain the information I send will be useful

□ Concern that information I send could be misunderstood

□ Other:

10. Do you currently use SMS (text communications using a cell phone) in your workplace for any of the following? Select all that apply.

□ Communication with patients and/or clients (for example, appointment reminders, lab results, care coordination, referrals)

□ Mass communications with or from your employer or other agencies

□ We do not use texting in the workplace

□ One-on-one communication with colleagues or co-workers (for example, consultation, sharing patient information, medication or prescription consultation)

□ Other:

15. During a public health emergency would it be better to receive a message by text/SMS or email?

□ Email

□ Text message

□ Both email and text message

□ Other:

16. Is there anything else you would like to tell us about your experience with texting, use of SMS by

public health or two-way vs one-way communications with public health?
